# 3,5-T2—A Janus-Faced Thyroid Hormone Metabolite Exerts Both Canonical T3-Mimetic Endocrine and Intracrine Hepatic Action

**DOI:** 10.3389/fendo.2019.00787

**Published:** 2020-01-08

**Authors:** Josef Köhrle, Ina Lehmphul, Maik Pietzner, Kostja Renko, Eddy Rijntjes, Keith Richards, João Anselmo, Mark Danielsen, Jacqueline Jonklaas

**Affiliations:** ^1^Institut für Experimentelle Endokrinologie, Charité Campus Virchow-Klinikum, Charité – Universitätsmedizin Berlin, Corporate Member of Freie Universität Berlin, Humboldt-Universität zu Berlin, and Berlin Institute of Health, Berlin, Germany; ^2^Institute of Clinical Chemistry and Laboratory Medicine, University Medicine Greifswald, Greifswald, Germany; ^3^MRC Epidemiology Unit, Institute of Metabolic Science, University of Cambridge, Cambridge, United Kingdom; ^4^Endocrinology Department, Hospital Divino Espirito Santo, Ponta Delgada, Portugal; ^5^Division of Endocrinology, Georgetown University, Washington, DC, United States

**Keywords:** thyroid hormone, 3,5-diiodothyronine, hypothyroidism, metabolome, anti-steatotic action, coffee metabolites, chemoluminescence immunoassay, deiodinase

## Abstract

Over the last decades, thyroid hormone metabolites (THMs) received marked attention as it has been demonstrated that they are bioactive compounds. Their concentrations were determined by immunoassay or mass-spectrometry methods. Among those metabolites, 3,5-diiodothyronine (3,5-T2), occurs at low nanomolar concentrations in human serum, but might reach tissue concentrations similar to those of T4 and T3, at least based on data from rodent models. However, the immunoassay-based measurements in human sera revealed remarkable variations depending on antibodies used in the assays and thus need to be interpreted with caution. In clinical experimental approaches in euthyroid volunteers and hypothyroid patients using the immunoassay as the analytical tool no evidence of formation of 3,5-T2 from its putative precursors T4 or T3 was found, nor was any support found for the assumption that 3,5-T2 might represent a direct precursor for serum 3-T1-AM generated by combined deiodination and decarboxylation from 3,5-T2, as previously documented for mouse intestinal mucosa. We hypothesized that lowered endogenous production of 3,5-T2 in patients requiring T4 replacement therapy after thyroidectomy or for treatment of autoimmune thyroid disease, compared to production of 3,5-T2 in individuals with intact thyroid glands might contribute to the discontent seen in a subset of patients with this therapeutic regimen. So far, our observations do not support this assumption. However, the unexpected association between high serum 3,5-T2 and elevated urinary concentrations of metabolites related to coffee consumption requires further studies for an explanation. Elevated 3,5-T2 serum concentrations were found in several situations including impaired renal function, chronic dialysis, sepsis, non-survival in the ICU as well as post-operative atrial fibrillation (POAF) in studies using a monoclonal antibody-based chemoluminescence immunoassay. Pilot analysis of human sera using LC-linear-ion-trap-mass-spectrometry yielded 3,5-T2 concentrations below the limit of quantification in the majority of cases, thus the divergent results of both methods need to be reconciliated by further studies. Although positive anti-steatotic effects have been observed in rodent models, use of 3,5-T2 as a muscle anabolic, slimming or fitness drug, easily obtained without medical prescription, must be advised against, considering its potency in suppressing the HPT axis and causing adverse cardiac side effects. 3,5-T2 escapes regular detection by commercially available clinical routine assays used for thyroid function tests, which may be seriously disrupted in individuals self-administering 3,5-T2 obtained over-the counter or from other sources.

**Graphical Abstract:**
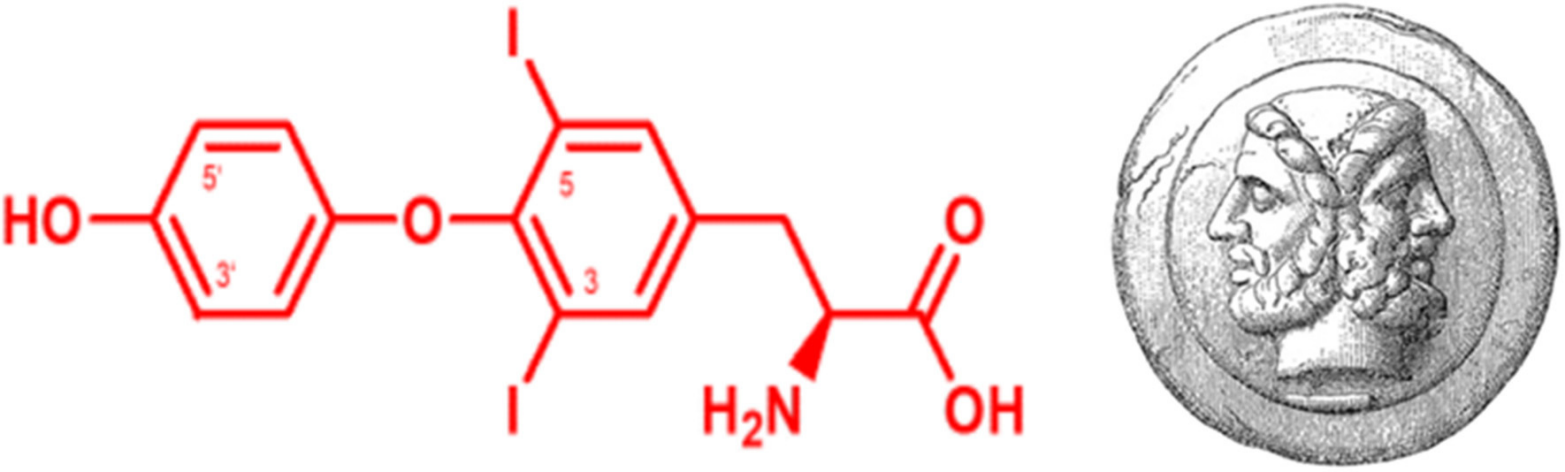
Structural formula of the “Janus-faced” THM 3,5-diiodo-L-thyronine (left), which has the same 3,5-iodine substitution pattern as its putative precursors L-T4 and L-T3 at the tyrosyl-ring, but lacks the iodine substitution in 3′-position of the phenolic ring which is essential for binding classical T3 -receptors and conveying canonical and non-canonical thyromimetic effects. The roman god Janus symbolized “duality” and “jani” were ceremonial gateways in ancient Rome typically used for symbolically auspicious entrances or exits. Janus-face (right) source: This image comes from the 4th edition of Meyers Konversationslexikon (1885–90). The copyrights have expired and this image is in the public domain. Wikimedia, Meyers_b9_s0153_b1.png.

## Hypotheses and Theory

- 3,5-T2 is an endogenous metabolite of thyroid hormones T4 and T3
- 3,5-T2 might represent the precursor of 3-iodothyronamine
- 3,5-T2 acts like T3 via canonical activation of T3 receptors albeit with lower potency
- 3,5-T2 exerts actions distinct from those of thyromimetically active T3
  - via mitochondrial targets
  - by its intrahepatic accumulation
  - by its intracrine mode of action
- 3,5-T2 formation and action might be altered in patients on T4 replacement therapy and causes adverse effects if abused.

## Introduction

### Endogenous Thyroid Hormones and Their Metabolites

A century of thyroxine research has led to the commonly held opinion in the thyroid hormone community that 3,3′,5,5′-tetraiodo-L-thyronine (L-Thyroxine, L-T4), which is solely produced by the thyroid gland, serves as a prohormone while T3 (3,3′5-triiodo-L-thyronine), in part secreted by the thyroid gland, is mainly generated in various extra-thyroidal tissues by either type 1 or type 2 deiodinase (DIO1, DIO2) selenoenzymes (1–3). T3 exerts the majority of known thyroid hormone (TH) effects at the tissue and cellular level, including hypothalamic and pituitary negative feedback regulation of the hypothalamus-pituitary-thyroid-periphery (HPTP) axis (4–6). Over the last years, some evidence has been presented that T4 also binds to cell membrane-located α*νβ*3 integrin receptors which exert rapid signaling via various kinase and intracellular pathways, especially in tumor cells and stem cells (7, 8). The T4 metabolite Tetrac, a deaminated side chain metabolite, present in human serum at concentrations similar to those of T3 (3, 9) antagonizes such T4 (and also T3) actions at the integrin receptor signaling. However, whether this has physiological relevance beyond these pharmacological approaches, mainly tested so far in cancer or stem cells, remains to be demonstrated. A further metabolite of T4 found in human serum, 3,3′,5′-triiodothyronine (reverse T3, rT3) has been studied over the last four decades because its production by the type 3 deiodinase (DIO3), also a selenoprotein, is increased under conditions when T3 production by DIO1 is impaired (3, 9) as well as during development in many tissues, when DIO2 activity is decreased (10). As rT3 is also degraded by DIO1, such conditions of high rT3 and low T3 (and T4) serum concentrations, summarized under the term “non-thyroidal critical illness” or “low T3 syndrome,” have caught the attention of clinicians attempting to use this constellation as a diagnostic or predictive readout. Up to now, no clear demonstration of rT3 function has been observed apart from its role during glial cell-mediated neuronal guidance in mammalian brain development (11). The initial hypothesis that rT3 might act as potent inhibitor of DIO1 or DIO2 during T3 formation (12, 13) could not be supported by *in vivo* experiments due to its short half-life and insufficient local concentrations (14). These observations did not support the hypothesis of rT3 acting as an autonomous regulator of extrathyroidal T3 formation under (patho-)physiological conditions.

### 3,5-T2 Is a Further Endogenous TH Metabolite With Thyromimetic Potency

**Figure 1:**
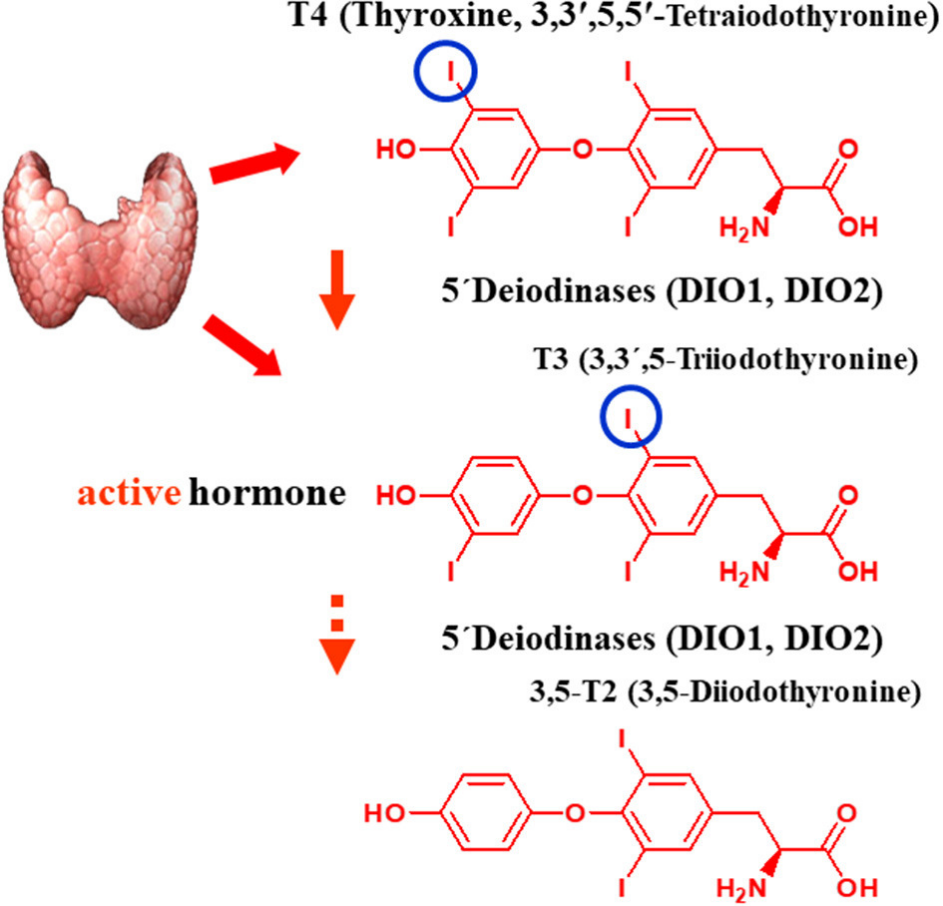
Postulated pathway of biosynthesis of 3,5-T2 from its putative precursors T4 and T3. The figure shows the structural formulas of L-T4, the prohormone, synthetized, and secreted by the thyroid gland, and its 5′-deiodination product L-T3, which is secreted in part by the thyroid gland (ca. 80%) or generated in extrathyroidal tissues by the two selenoenzyme 5-deiodinase type 1 or type 2, which both remove the 5′-iodine atom of L-T4 in a reductive two-substrate reaction with a so far unknown physiological cofactor. Indirect evidence mainly from *in vivo* experiments in rodents suggests that 3,5-T2, a physiologically active endogenous thyroid hormone metabolite, is formed by a further 3′-deiodination reaction also catalyze by these (one of these) two deiodinase enzymes.

The TH metabolite 3,5-T2, possibly formed from its precursor T3 (Figure 1), has recently attracted great interest for several reasons (3, 9, 15). 3,5-T2 has been considered the main biological active metabolite of T3, formed via further phenolic ring deiodination from T3 (Figure 1). The TH metabolite 3,5-T2 is found in blood and at even higher concentrations in several tissues. Various groups have demonstrated that 3,5-T2, in addition to its thyromimetic action at the classical T3 receptors at high concentrations, exerts rapid direct effects on mitochondria (6, 16–19), which might be beneficial in terms of stimulation of oxygen consumption, increased hepatic, and muscular lipid metabolism—all of these effects appear as potentially favorable in global attempts to combat steatosis in liver and other tissues.

Therefore, we intended to test several hypotheses using clinical experimental data, animal experiments, and cell culture approaches as well as recently developed novel analytical tools such as the sensitive chemoluminescence immunoassay (CLIA), highly specific for 3,5-T2 detection in human serum (20). We aimed to test the hypotheses that

- 3,5-T2 is formed from T4 and T3 as its precursors via deiodinase reaction,
- 3,5-T2 circulates in human serum, and that various pathophysiological as well as experimental conditions lead to alterations in 3,5-T2 serum concentrations.
- Furthermore, that effects of administration of physiological and pharmacological doses of 3,5-T2 in mouse models will provide information on its potential thyromimetic activity as well as for its potentially beneficial antisteatotic action.
- Moreover, analyses using various *in vitro* cellular models might convey an insight into postulated mechanism(s) of action of 3,5-T2 at the level of gene expression and/or mitochondrial energy metabolism.
- 3,5-T2 causes adverse effects if abused and/or overdosed.

This series of clinical, translational and basic science studies testing these options resulted in a complex picture of various actions of 3,5-T2. The outcome was variable depending on concentrations applied, experimental models used, and analytical tools applied so far. 3,5-T2 turned out to be an ambiguous, *Janus*-type endogenous thyroid hormone metabolite (THM) with beneficial and adverse biological effects. Analytical tools available, cannot yet dispel previously reported controversies regarding its baseline concentrations in human serum and the variations in concentration caused by pathophysiological states.

Currently, the thyroid community and patients discuss why a fraction of patients on established L-T4 replacement therapy, due to thyroidectomy or autoimmune thyroid disease, are subjectively discontent with this therapeutic regimen compared to individuals with intact thyroid, albeit their thyroid function test are in the reference range. We hypothesize that endogenous production of 3,5-T2 in some patients dependent on oral L-T4 might be less than that in individuals whose intact thyroid is the source of T4, and thus an inadequate 3,5-T2 concentration might contribute to this discontent based on its distinct thyromimetic activity compared to T3.

## 3,5-T2 Concentrations in Human Serum

### Immunoassays for 3,5-T2 Using Polyclonal Antisera Reveal Divergent Concentrations

Following the detection of the thyromimetically active T3 in human serum and its production from T4 in athyreotic humans supplemented with L-thyroxine (21, 22), attempts were also made to quantify concentration of 3,5-T2 in human serum and tissues. Most of these studies were feasible after the development of specific radioimmunoassays, based on poly- or monoclonal antibodies in the Seventies of the last century, and supported extrathyroidal formation of 3,5-T2, which had been previously demonstrated using radioisotope-labeled TH in humans, experimental animal models, and *in vitro* systems [see recent reviews: (3, 9)]. Initially, various groups attempted to quantify 3,5-T2 under physiological and pharmacological conditions and in various disease states, mainly using radioimmunoassays based on specific polyclonal antibodies generated against 3,5-T2 conjugates in various animal models. The assays developed during the 1980s were sensitive enough to detect 3,5-T2 in unextracted serum samples directly, as summarized in Table 1. In contrast to rather precise and narrow concentration ranges detected for total T4, total T3, and total rT3 in human serum, the concentration ranges for 3,5-T2 showed remarkable differences between various assays used, exceeding more than one order of magnitude from 4 to almost 200 pM/L. This is in sharp contrast to concentrations reported for the other THM formed from either T3 via 5-deiodination at the tyrosyl- ring (3,3′-T2), or from rT3 generated by phenolic ring deiodinaton (3,3′-T2) or the rare T2 metabolite 3′,5′-T2 generated from rT3 via tyrosyl-ring deiodination. Reported serum concentrations covered a very narrow concentration range between 36 and 68 pM for 3,3′-T2 (see Table 1) (23–29, 32–34). Authors discussed this point, but no clear explanation or hypothesis was put forward to explain this peculiar observation atypical for the usually highly precise immunoassays for THM. The variable results might be explained by technical difficulties in reproducibly synthesizing and purifying tyrosyl-ring mono-labeled tracers required for these competitive immunoassays, as it turned out to be very difficult to avoid additional radioactive labeling of the phenolic ring of 3,5-T2, which would yield a T3-analog tracer. Only complex synthesis procedures different from the typical Chloramine T labeling protocols used for T3, rT3, or T4 yielded clean, radioactive tracers solely mono-labeled at the tyrosyl-ring (38). Thus, reports on serum 3,5-T2 concentrations determined by immunoassay methods need to be interpreted with caution.

**Table 1:** Serum concentrations reported for 3,5-T2 and 3,3′-T2 over the last 4 decades using different analytical techniques (immunoassay formats, mass spectrometry).

**References**	**Assay type**	**Serum 3,5-T2 (pM)**	**Serum 3,3^**′**^-T2 (pM)**	**Species**
Richards et al. (23); Richards et al. (24); Richards et al. (25)	LC/MS-MS	<5	**41** ± 8	Human
Lehmphul et al. (20)	CLIA	**290** ± 10	–	Human
Gu et al. (26)	LC/MS-MS	–	**52** ± 16	Human
Jonklaas et al. (27)	LC/MS-MS	–	**12.2–28.2**	Human
Moreno et al. (28); Pinna et al. (29)	RIA	**4.6** ± 0.4	**68** ± 21	Rat
Pinna et al. (30)	RIA	**16.3** ± 6.4	–	Human
Engler and Burger (31)	RIA	**7.6** ± 3.4	–	Human
Jaedig and Faber (32)	RIA	**70**	**42**	Human
Kirkegaard et al. (33)	RIA	**105** ± 51	**36** ± 15	Human
Nishikawa et al. (34)	RIA	**196** ± 36	**67** ± 36	Human
Pangaro et al. (35)	RIA	**82** ± 4	–	Human
Maciel et al. (36)	RIA	**139** ± 67	–	Human
Meinhold and Schürnbrand (37)	RIA	**100**	–	Human

*Bold values give mean serum concentrations reported by authors*.

A similar problem exists also with respect to the purity of 3,5-T2 standards used in these immunoassays, as commercial preparations available now and in the past frequently were significantly contaminated with T3 in concentrations high enough to provide interference by T3 serum concentrations, which are typically an order of magnitude higher than those of 3,5-T2 in the 3 nM concentration range.

A second difficulty still not clarified is the possibility of a distinct binding of 3,5-T2 to (yet unknown) serum distributor or transporter proteins, in comparison to T3 and T4 which are known to preferentially bind to the classical thyroid hormone distributor proteins thyroxine-binding globulin (TBG), transthyretin (TTR), and albumin. If residual binding to any serum distributor protein for 3,5-T2 would not be eliminated by incubation conditions or additives releasing 3,5-T2 from this binding, the assumptions of adequate equilibrium competition during incubation time and separation of free ligands from those bound to the antibodies would be compromised. Irrespective of these difficulties, various research groups set out to analyze 3,5-T2 concentrations in human serum in reference populations and individuals with altered thyroid function as well as disease states, but no uniform picture emerged and even observations of concentration changes in clinical hyperthyroidism vs. hypothyroidism were not uniform. Of interest was the observation that tissue levels of 3,5-T2, for example in the rat brain, were much higher than expected from serum concentrations and reached values in the range of those of T4 and T3, as clearly shown by Pinna et al. (29), who also observed changes in 3,5-T2 concentrations of various rat brain regions after administration of drugs used in neurology and psychiatry. Similarly, a notable hepatic accumulation of 3,5-T2 was observed after administration of exogenous 3,5-T2 in a mouse model (39) suggesting a role as a tissue-resident “intracrine” hormone.

### A Chemoluminescence Assay Based on Monoclonal Antibodies Detects 3,5-T2 Serum Concentrations Not Directly Related to Classical Parameters of Thyroid Hormone Status

**Figure 2:**
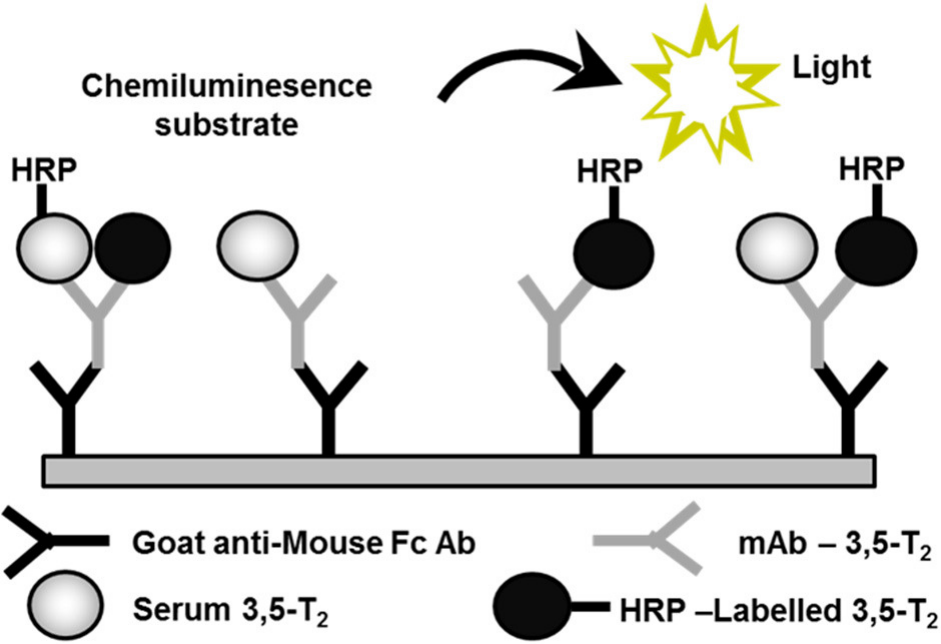
Assay principle used for the development of a monoclonal antibody-based chemoluminescence immunoassay (CLIA). This method allows to detect 3,5-T2 in human serum at low concentrations and under various pathophysiological conditions (20, 40). HRP, horse-radish peroxidase; mAB, monoclonal antibody; Fc Ab, Fc-fragment of a goat antibody recognizing mouse mAb.

**Figure 3:**
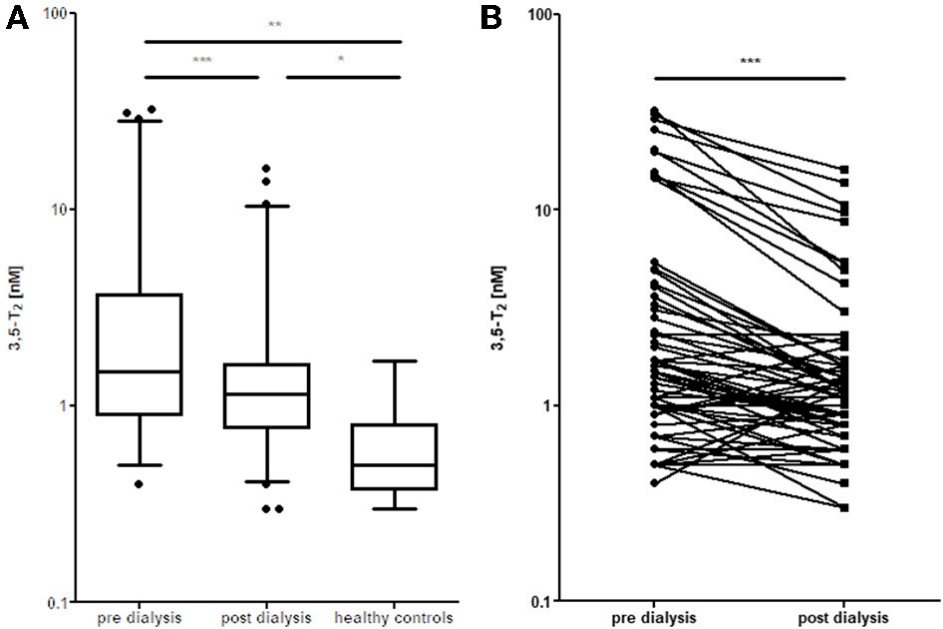
Elevated 3,5-T2 concentrations in serum of dialysis patients. **(A)** Decrease in serum 3,5-T2 concentrations after dialysis, but still elevated compared to healthy controls (*p < 0.05; **p < 0.01; ***P < 0.001). **(B)** Comparison pre vs. post dialysis; paired *t*-test, Wilcoxon signed rank test (Gaussian approximation) (***P < 0.001) (43).

We set out to develop a monoclonal antibody-based CLIA, which allows detection of 3,5-T2 in human serum at low concentrations and under various pathophysiological conditions. Figure 2 illustrates the assay principle used (20, 40). Monoclonal antibodies recognizing 3,5-T2 with high specificity and very low cross-reactivity to other THM present in human serum were incubated in a micro-titer plate assay format, wells were coated with goat anti-mouse Fc antibody, and serum containing 3,5-T2. After incubation and various washing steps, horseradish peroxidase labeled 3,5-T2 competed with serum 3,5-T2 for binding to the anti-3,5-T2 monoclonal antibodies, and after equilibration of this competitive arrangement, chemiluminescence substrate was added to produce light and quantifying HRP labeled 3,5-T2 tracer vs. endogenous 3,5-T2. Using this assay, validated according to state-of-the-art technology with respect to assay stability, specificity, and reproducibility, we detected serum 3,5-T2 concentration in reference populations around 290 pM. Application of this immunoassay to analyze 3,5-T2 concentrations under various clinical conditions yielded unexpected results. First of all, 3,5-T2 concentrations in hypothyroid or hyperthyroid patients were not different from those of the reference population, while total T4 and T3 showed expected changes. We did not observe gender- or age-dependent variations, nor were concentrations correlated with BMI. However, thyroidectomized patients (after thyroid cancer diagnosis) showed slightly elevated serum concentrations compared to the healthy reference population (20), an observation to be confirmed by LC-LIT-MS/MS/MS (LC-LIT-MS^3^) analysis. Patients with post-operative atrial fibrillation (POAF) also had higher 3,5-T2 concentration than a similar group of cardiology patients without POAF (41). Of interest was the observation that, in a group of intensive care unit (ICU) patients, non-survivors had significantly higher 3,5-T2 concentrations than those who survived (42). This was unexpected considering the low T3 concentrations in those patients in context of the hypothesis that 3,5-T2 would be a direct product of T3 and potentially lowered under conditions of decreased DIO1 (or DIO2) activity. Even more surprising was the observation of remarkable changes of 3,5-T2 concentrations, which were increased by more than an order of magnitude in patients on pre-dialysis compared to post-dialysis, with demonstration of even lower concentrations in healthy controls (20). Figure 3 indicates this remarkable concentration range and increase in 3,5-T2 concentrations, which, with respect to the amplitude observed, is much higher than reported for any of the changes in T4, T3, rT3, or 3,3′-T2 concentrations so far. This data and the observation in ICU patients would be compatible with the hypothesis that impaired renal elimination of 3,5-T2 leads to its accumulation in serum or, alternatively, impaired renal function might contribute to elevated 3,5-T2 production. Such hypotheses need to be tested in appropriate prospective studies. Still those observation based on CLIA analysis of serum concentrations of 3,5-T2 require confirmation by LC-LIT-MS^3^ analysis.

**Figure 4:**
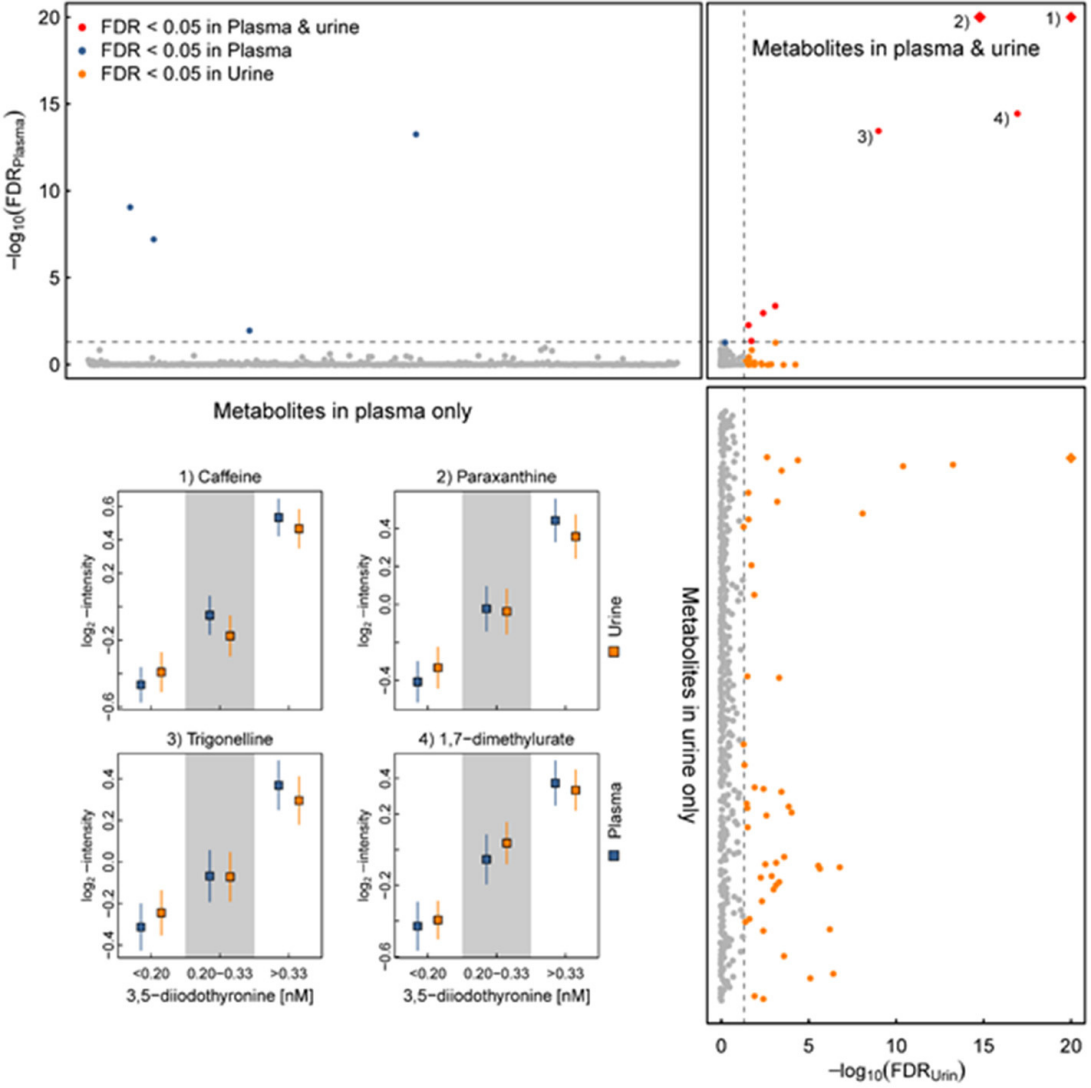
Serum 3,5-T2 concentration and multifluid (urine and plasma) metabolomics in healthy individuals of the SHIP-Trend cohort. **(Upper)** *P*-values (y-axis) for plasma metabolites (x-axis) after correction for multiple testing (false discovery rate—FDR) comparing participants with low serum 3,5-diiodothyronine (3,5-T2) concentrations (<0.20 nM) with participants with high serum 3,5-T2 concentrations (>0.33 nM). Values have been transformed by –log_10_ to ease visualization of small values. **(Upper right)** Opposing FDR-values for the same comparison for metabolites measured in plasma (y-axis) and urine (x-axis). **(Left)** Similar to upper panel but showing the results for metabolites measured in urine only. Darker colors indicate metabolites meeting the threshold for statistical significance (FDR < 0.05) and red indicates metabolites consistently associated in both fluids. Inset: Predicted least-square means with 95%-CIs from linear regression models for four metabolites selected across the three approximately equally-sized groups of participants divided according to serum 3,5-T2 concentrations (44). All *p*-values were derived from a linear regression model treating serum 3,5-T2 concentrations as categorical exposure and metabolites as outcome, controlling for age, sex, waist circumference as well as serum thyroxine and thyrotropin concentrations. Blue—plasma and orange—urine.

During our attempts to establish a reference range for 3,5-T2 in a healthy population, we analyzed 3,5-T2 concentrations in a subset of the population-based Study of Health in Pomerania (SHIP-Trend) including 761 euthyroid participants. The analysis comprised associations with various anthropometric and clinically relevant parameters. Surprisingly, one third of the study population had 3,5-T2 serum concentrations determined by CLIA below the limit of quantification but above the limit of detection, yielding a median serum concentration of 0.24 nM with right-skewed distribution. Associations of the 3,5-T2 concentrations with various clinical chemistry parameters were moderate. Of special interest was, similar to the previous studies, the lack of a clear association with serum T4 and/or T3 concentrations (44, 45). In a follow-up study of the same cohort, 3,5-T2 serum concentrations were analyzed with respect to urine metabolites analyzed by ^1^H-NMR spectroscopy (45–48). Again, very few individuals had remarkably elevated 3,5-T2 serum concentrations that might be suggestive of underlying disease, although health status could not be traced back due to the population-based study using anonymized sera. However, 3,5-T2 serum concentrations were remarkably positively associated with various urine metabolites. More detailed metabolome analyses revealed that these metabolites represent a “signature of coffee consumers.” Among these urinary metabolites are trigonelline, pyroglutamate, and hippurate. This observation is the first one to link a THM to coffee consumption, and so far, no clear-cut hypothesis has been tested to challenge a potential causal relationship (45, 46). However, indirect evidence might support the assumption that hepatic accumulation of 3,5-T2 (see below) may alter hepatic (and/or renal) metabolism of components consumed with (caffeinated and decaffeinated) coffee and thus even might provide a link between beneficial antisteatotic effects ascribed to 3,5-T2 and also to coffee consumption (44–48) (Figure 4). Table 2 summarizes the observations made in various pathophysiological states with respect to altered 3,5-T2 concentration in human serum, as determined by the monoclonal antibody-based CLIA.

**Table 2:** Summary of serum 3,5-T2 concentrations in humans observed under physiological (a) and pathophysiological (b) conditions.

**a) Physiological conditions:**
• Serum 3,5-T2 and 3T1AM concentrations as analyzed by specific mAb-based CLIA do not mirror the dynamics of T3 (or T4) after substitution with T4, T3, or T3-sulfate in healthy individuals or hypothyroid patients • Inter-individual differences in 3,5-T2 and 3T1AM serum concentrations • Remarkably stable individual 3,5-T2 and 3T1AM serum concentrations • Discrepancy between 3,5-T2 serum concentrations determined by CLIA and LC-MS/MS • No clear correlation to TSH, T4, and T3 concentrations • No evidence from these studies supporting the postulated metabolic pathway in humans: T4 → T3 → 3,5-T2 → 3T1AM • Serum 3,5-T2 concentrations are correlated to trigonelline, hippurate, and 3-aminoisobutyrate concentration in urine of healthy individuals (“coffee signature”).
**b) Elevated serum concentrations of 3,5-T2 in patients:**
• Sepsis • Non-survivors of ICU • Postoperative atrial fibrillation (POAF) • Impaired renal function • Oral T4 supplementation.

## Biosynthesis−3,5-T2 May Not be Generated Directly From its Putative Precursors T4 and/or T3 in Humans

The discovery and demonstration of sequential mono-deiodination pathways catalyzed by the three deiodinase isoenzymes using tetra, tri-, di-, and mono-iodinated iodothyronines as substrates has led to the assumption that 3,5-T2 formation *in vivo* occurs via 5′-deiodination of T3 catalyzed by either DIO1 or DIO2, while not much evidence was available that 3,5-T2 would be a direct secretion product from iodinated thyroglobulin. The existence of 3,5-T2 in thyroglobulin has not been clearly demonstrated (49, 50) and appears biochemically improbable considering that TPO catalyzes coupling of mono- and/or di-iodinated tyrosine residues to yield either T4 or T3, while no such coupling was observed between diiodotyrosine and iodine-free tyrosine in reports available. Formation of iodothyronines outside of the thyroid gland has not been reported in humans or mammalian organisms while various aquatic life forms synthesize T4 without presenting the highly evolved follicular structure of thyroid glands in vertebrates (51). Circumstantial evidence using the DIO1 inhibitor PTU in isolated rat mitochondria or perfused rat liver led to the assumption that 3,5-T2 is a logical deiodinase product of T3 (16, 52), but various attempts to demonstrate such a reaction *in vitro* failed to support this hypothesis. In contrast, *in vivo* data from experimental animal models and human studies, where PTU has been administered, resulted in lower 3,5-T2 concentrations compared to appropriate controls. However, these studies were also based on immunoassay serum analytics.

**Figure 5:**
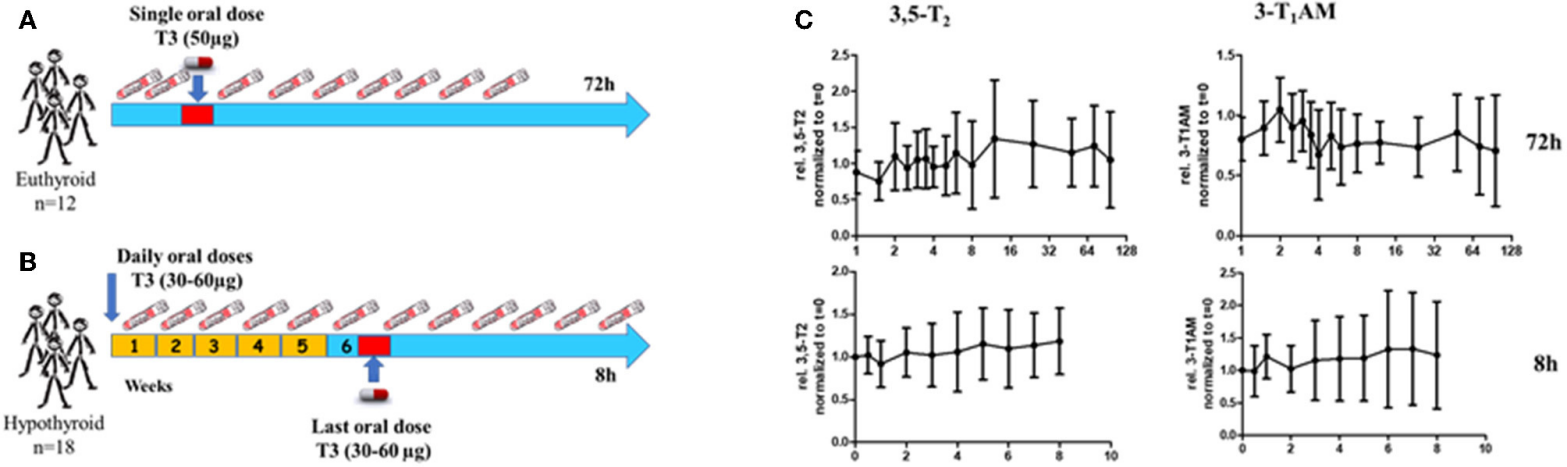
Experimental paradigms to test whether 3,5-T2 is formed from T4/T3 in humans. **(A)** Twelve euthyroid healthy volunteers received a single oral dose of L-T3 (50 μg) and blood samples were drawn during the following 72 h (53). **(B)** Eighteen hypothyroid patients were switched from T4 to daily doses of 15 μg Liothyronine (T3) at week 1. Their daily T3 dose was then increased to 30 μg after week 2 and increased as necessary up to 60 μg T3 to maintain a normal serum TSH. Serum samples were drawn weekly and also for a kinetic study during the 8 h after the final T3 dose was administered in week 6 (54). **(C)** Serum concentrations (relative to individual concentrations at *t* = 0 h) for the two kinetic studies (see above) administering Liothyronine (T3) to euthyroid volunteers (upper panel) and hypothyroid patients (lower panel). Individual serum concentration data are provided in Supplementary Data Sheet 1. No clear increase was found in serum 3.5-T2 concentration in both paradigms and also no increase was observed for 3-T1AM, the postulated product generated from 3,5-T2 via combined deiodination and decarboxylation).

In the light of marked variations of serum concentrations of 3,5-T2 in humans as determined by the monoclonal antibody-based CLIA, we set out to test whether 3,5-T2 is formed *in vivo* in humans if T4 and/or T3 were administered. Several experimental paradigms were tested for acute or chronic 3,5-T2 formation from T4 and/or T3. Figure 5A shows the experimental approaches used by Jonklaas et al. (53), who administered a single dose of T3 (50 μg) to 12 euthyroid individuals and sampled serum over 72 h. Assuming T3 as precursor of 3,5-T2, one would expect an increase of 3,5-T2 concentration after T3 administration. In a second paradigm (Figure 5B) (54), T3 was administered at concentrations between 30 and 60 μg to hypothyroid patients, who at baseline were substituted with T4. Their T4 was discontinued and they were instead treated with a daily dose of 15 μg of T3. Subsequently the T3 dose was increased by 15, 30, or 45 μg to replace T4 over a period of 4 weeks aiming for non-suppressed TSH. Blood samples were drawn at baseline and then weekly during T3 treatment. Blood samples were also drawn hourly for 8 h after the final T3 dose was given (Figure 5B). In a third paradigm, we determined 3,5-T2 serum concentrations (data not shown) in 10 thyroidectomized patients who were treated with L-T4 or L-T3 to target their serum TSH to the reference range of 0.5–1.5 mU/L for at least 30 days, in order to test whether T3 and T4 can lead to similar TSH concentrations in the reference range (55). In the fourth study, 3,5-T2 concentrations were determined (data also not shown) in sera of volunteers receiving 100 μg of T3 sulfate, an endogenous THM, which might act as reservoir or precursor to rapidly yield T3 liberated by ubiquitous sulfatase enzymes (56). T3 sulfate is formed during enterohepatic circulation of TH, and 3,3′-T2 sulfate is a metabolite generated by the fetus and transferred to maternal circulation during pregnancy (57, 58). Again, rapid formation of T3 from T3 sulfate might lead to elevated production of 3,5-T2 if T3 and/or T3 sulfate would be precursors of 3,5-T2. While analysis of T3 in serum for all four paradigms revealed the expected kinetic profiles and TSH responses (53, 55, 56), the 3,5-T2 concentration profiles found (Figure 5C) were unexpected. In all of the above mentioned four paradigms, irrespective of T4, T3, or T3 sulfate administration, significant changes of 3,5-T2 serum concentration as determined by mAB-CLIA were not observed [and also no changes in concentrations of 3-T1AM, also determined by monoclonal antibody CLIA (59), were found]. 3,5-T2 had been postulated as a potential precursor of the biogenic amine 3-iodo-thyronamine (3-T1AM), possibly enzymatically generated from 3,5-T2 by a combination of deiodination and decarboxylation (60, 61).

**Figure 6:**
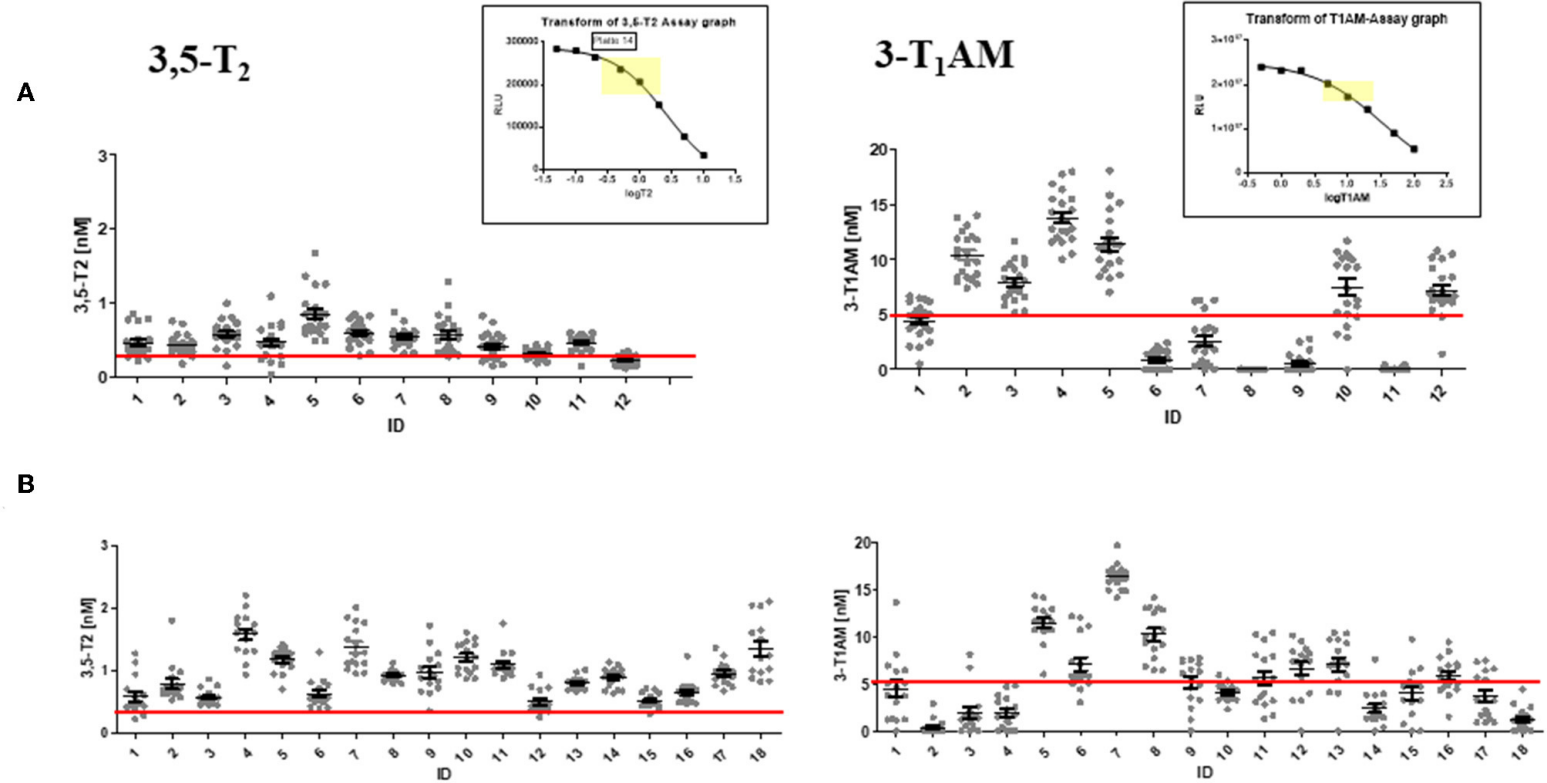
Remarkably stable individual 3,5-T2 serum concentrations during several hours and days of sampling time but significant inter-individual differences. Graphs show absolute serum concentrations of 3,5-T2 (left) and 3-T1AM (right) for each individual receiving Liothyronine in the two kinetic studies presented in Figures 5A,B. Upper panel **(A)**: 12 euthyroid volunteers with blood drawn over 72 h; lower panel **(B)**: 18 hypothyroid patients with blood drawn over 8 h. Inter-individual differences for 3-T1AM serum concentrations seem to show higher variations than those for 3,5-T2, and variations over time might be more pronounced than those of 3,5-T2. Individual serum concentration data are provided in Supplementary Data Sheet 2. Insets show typical standard curve for 3,5-T2 and 3-T1AM CLIA standard curves, with reference range marked in yellow. The red line represents the preliminary reference concentration for healthy controls.

Together, these studies might indicate that 3,5-T2 is not directly generated from serum T3 and its serum profile does not mimic the transient increases observed for T3 after its exogenous administration in humans. Several interpretations can be put forward to explain this situation: (i) 3,5-T2 is not a direct product of T3, or (ii) increases in T3 or T3-sulfate concentration in serum are not reflected by corresponding coincident or delayed 3,5-T2 concentration profiles, (iii) but 3,5-T2 might be formed intracellularly in various tissues; (iv) the half-life of 3,5-T2 may be too short to lead to accumulation of this metabolite in human serum after T3, T3-sulfate^1^, or T4 administration; (v) the monoclonal antibody-based immunoassay for 3,5-T2 may recognize also additional cross-reacting compounds not formed after *in vivo* application of T4, T3, or T3-sulfate. However, these observed patterns in sera of these four studies do contradict the above-mentioned observations that higher 3,5-T2 concentrations were observed in thyroidectomized patients on oral T4 replacement therapy (20, 62). Figure 5C shows the serum profiles of 3,5-T2 found after T3 administration in healthy controls and hypothyroid patients, respectively, in the two Jonklaas studies (53, 54). Assay results had to be normalized to time zero 3,5-T2 concentrations to generate the kinetic profile. Of note are quite distinct individual 3,5-T2 concentrations both in healthy individuals and hypothyroid patients that were not affected by T3 administration, with intra-individual 3,5-T2 serum concentrations that remained remarkably stable during several hours and days of sampling time (Figure 6).

## 3,5-T2 Serum Concentrations Are Remarkably Stable and Distinct Between Individuals

3,5-T2 concentrations in individuals exhibit remarkable stability over sampling time. Figure 6 illustrates distribution of 3,5-T2 concentrations in various individuals over sampling time for several hours and days. Figure 5C represents the analysis of 3,5-T2 concentrations in healthy individuals (*n* = 12), to whom a single dose of 50 μg T3 was administered, and blood samples were taken regularly over 72 h (53). Most individuals exhibit very narrow concentration ranges with small variations over sampling time while individuals differ among each other in their 3,5-T2 concentration in serum. Figure 5C also shows similarly analyzed 3,5-T2 concentrations in 18 hypothyroid patients, who received T3 doses between 30 and 60 μg total, following discontinuation of T4 treatment (54). T3 was dosed to bring TSH concentrations into the euthyroid reference range. Compared to healthy individuals, inter-individual differences between 3,5-T2 concentrations were more pronounced; nevertheless, each individual again showed rather stable 3,5-T2 concentrations over time, considering that, as illustrated above in Figure 6, no significant changes in 3,5-T2 concentrations were observed after T3 administration. Similar patterns of rather stable individual 3,5-T2 concentrations were observed in a drug study on volunteers, who underwent food withdrawal and refeeding protocols while treated with an experimental drug (J.K. et al., unpublished data). But again, explicit inter-individual differences were observed between the volunteers, while 3,5-T2 concentrations remained fairly constant in each individual over several circadian phases, food withdrawal and refeeding cycles, and experimental drug administration. Only one individual was exceptional in exhibiting rather pronounced variations of 3,5-T2 for unknown reasons (unpublished data, van Vliet and Köhrle). Unfortunately, no residual, sufficiently large serum sample volumes (additional 200 μL) were available to repeat those studies using the recently developed new LC-LIT-MS^3^ analysis of THM (25).

Taken together, the determination of 3,5-T2 concentrations by mAb-based CLIA in experimental human studies involving administration of T4, T3 alone, as well as T3 sulfate, or an experimental drug did not provide evidence that the postulated metabolic pathway of T4 → T3 → 3,5-T2 deiodination could be supported in analyses after short or medium-term interventions in humans. Thus, it remains to be studied whether 3′(5′)-deiodination of T3 to 3,5-T2 occurs in any human tissues and can be measured in serum in healthy volunteer individuals or hypothyroid patients requiring TH substitution.

## Alternative LC-MS-Based Approaches Are Needed to Determine Concentration of Thyroid Hormones and Their Metabolites in Human Serum

Over the last years, the discovery of various THM with either lower iodination grade or modifications at the functional residues of iodothyronines, that is the 4′-hydroxy group conjugation or modification of the alanine side chain, and subsequent studies on the potential biological relevance of various THM (see Graphical Abstract) has led to various attempts to quantify the whole spectrum of iodinated and iodine-free metabolites [“the thyronome” profile; (3)] using liquid chromatography—tandem mass spectrometry (LC-MS/MS) techniques [for a recent review see (23)]. Initial attempts to quantify T4 and its metabolites by GC-MS did not gain acceptance for clinical diagnostics. The MS-based analysis of THM is faced with several challenges: (i) first of all, should total and/or free concentrations be determined in whole blood, serum, plasma, or other body fluids like saliva, cerebrospinal fluid, or urine? (ii) How can THM be quantitatively analyzed considering their extremely high affinity binding to various distributor proteins contained in plasma, such as TBG, TTR, albumin, or APO-lipoprotein B100? (iii) Which chromatographic procedure faithfully will separate isobaric tri-, di-, or mono-iodinated structural isomers of T4 metabolites? (iv) Is the sensitivity of combined LC-MS sufficiently high to quantify in a single run nanomolar total T4 and T3 concentrations and low picomolar concentrations of mono- or di-iodinated THMs? (v) Which influence will matrix composition of plasma or serum from healthy individuals vs. sick patients exert, and can this be adequately standardized to accurately quantify the concentrations of the THM spectrum in clinical practice?

Appreciating the major progress made during the last two decades in mass spectrometry-based analytics of steroid hormones (63–66), several groups attempted to draw level with those laboratories in the TH community also. However, various practical and technical issues related to above-mentioned challenges of TH physiology and pathophysiology hampered rapid progress and success in quantification of total and free THM concentrations. While meanwhile several laboratories developed procedures to accurately determine total T4 and total T3 in human plasma or serum and largely agree on concentrations ranges (25, 67–73), this consensus has not been achieved yet for most of the other TH metabolites, which occur at much lower concentrations. Analytical procedures typically work well with buffer solutions, *in vitro* reaction mixtures, as well as with samples of “simple” matrix composition, while reported concentrations in human or experimental animal serum, plasma, and tissue show wide variations and marked insufficiencies in terms of method validation, standardization, and quality assessment parameters [see (23, 24, 74)]. Most groups reporting data employed liquid-liquid or solid-phase extraction procedures with or without previous protein precipitation to enrich and purify THM profiles from matrix components. Nevertheless, sample recoveries, process efficiencies, and matrix effects were not convincing in most of the cases published, if reported at all. Although the teams used the principle of isotope dilution technique, as far as isotope-labeled standards for the individual thyronine metabolites are available, no uniform picture has evolved yet. It has become apparent that for accurate quantification of various THM, use of only one or two stable isotope-labeled internal TH standards is not sufficient due to the very distinct binding of THM, which are highly hydrophobic but still polar amino acid derivatives. This tight binding not only relates to their distribution proteins in blood, cells or tissues but especially also to surfaces of plastic material required during pre-analytical sample work and subsequent MS quantification. Recently, two groups reported for the first time 3,5-T2 serum concentrations determined by LC-MS. Lorenzini et al. (75), observed a mean concentration range for 3,5-T2 in human serum of 78 ± 9 pM, while our own results (25) indicated that most human sera analyzed for 3,5-T2 have lower than 5 pM 3,5-T2 concentrations, which is the lower limit of quantification in our method. The fact that Lorenzini et al. probably report higher LC-MS-based 3,5-T2 concentrations is due to the contamination of the 13^C^9-15^N^-3,5-T2 internal standard, with “natural” unlabeled 3,5-T2. While we used very high dilution of this internal isotope labeled standard once we discovered this problem, Lorenzini et al. who used the same supplier for this material, probably included this “exogenously added 3,5-T2” in their analysis while adding much higher undiluted internal standard concentrations to their samples. A surprising outcome of both our own and the Lorenzini study, however, is that MS-based analysis of 3,5-T2 concentration in sera of healthy individuals is significantly lower than concentrations reported by our above-mentioned CLIA based on monoclonal antibodies. At this point, no explanation for the divergent results is possible yet, but needs to be considered during future analysis and further improvement of analytical methods for concentrations determination of 3,5-T2 in human and animal serum (25). The method used by Richards et al. involved a completely novel pre-analytical approach involving single step solid-phase delipidation, avoiding multiple use of plastic material during sample workup and employing silanized glass vessels during this procedure. This sample work-up was then combined with isotope dilution, LC-positive ion electron spray multiple reaction monitoring (MRM) linear ion trap LIT-MS^3^, which allowed clear separation of isobaric tri- and di-iodinated iodothyronines and resulted in acceptable signal-noise ratios for the LIT-MS^3^ scans leading to limits of quantification in the low picomolar range. Assay accuracies, precisions, and process efficiencies with quality assessment parameters were obtained which are requested by authorities for analytical procedures utilized in clinical diagnostics. Application of this procedure allows for simultaneous, pre-analytical extraction and mass-spectrometry quantification of T4, T3, rT3, 3,3′T2, and 3,5-T2 in one single run using 200 μl serum (or less, if the two di-iodinated thyroid hormones are not required for these analytics). Major work is needed to clarify the differences between mAb-based immunoassays and MS-based analytics. Previously, other groups already reported major deviations between immunoassay- and LC-MS-based methods, if T3, rT3, or 3,3′T2 were analyzed in human serum (27, 76, 77). Application of this new method for determination of free TH concentrations will require significantly more work on method development and quantification.

Using the monoclonal antibodies employed in the CLIA methods, a series of immune extraction experiments from native or spiked human serum was performed to clarify the differences found in 3,5-T2 concentrations in human serum using either the CLIA or LC-MS method (unpublished work). Human serum aliquots were either used as prepared after blood drawing or after spiking with analytes of interest. Aliquots with known amounts of either 3,5-T2 or 3-T1AM sera were incubated for equilibration at room temperature for 1 h, then highly specific mAbs against 3,5-T2 (20) or 3-T1AM (59) were added in form of beads covalently bound to these antibodies, then sera were incubated overnight at 4°C, serum was removed by centrifugation, and antibody beads were recovered. Beads were washed several times, and bound THM were eluted with methanol and analyzed either by LC-MS/MS (25) or TOF-LC-MS. Evaluation of extracts from mAb beads by LC-MS analysis (25) revealed that the 3,5-T2 antibody efficiently extracted 3,5-T2 from serum as expected but did not bind and extract spiked 3-T1AM or other endogenous THM. In contrast, the mAb against 3-T1AM, also coupled to beads, extracted spiked 3-T1AM from serum, but no spiked 3,5-T2, no other THM and surprisingly not any “endogenous” 3-T1AM was found in the extracted eluates (Table 3) (J.K. et al., unpublished data). Careful analysis by TOF-LC-MS did not provide information on significant enrichment or extraction of any unknown serum components cross-reacting either with the 3,5-T2 or the 3-T1AM mAbs to a measurable extent. Thus, a carefully controlled immune-extraction approach of native or THM spiked serum did not reveal, why 3,5-T2 concentrations determined by mAB-based CLIA were so much higher than those endogenous 3,5-T2 concentrations determined by LIT-MS^3^ and the recently described one-step pre-analytical extraction method.

**Table 3:** Ligand binding and cross reactivity of monoclonal antibodies used in 3,5-T2 and 3-T1AM CLIA as analyzed by immune extraction.

**mAb**	^**2**^H_**4**_-3-T_**1**_AM^*^	^**13**^C_**9**_-^**15**^**N-3,5-**${\text{T}}_{2}^{*}$	**3,5-T_**2**_**	**T_**3**_**	**rT_**3**_**	**T_**4**_**	**3-T_**1**_-AM**
(3-T_1_-AM)	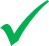	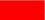	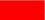	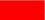	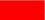	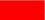	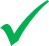
(3,5-T_2_)	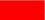	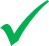	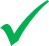	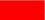	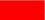	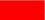	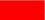

*Ligand binding specificity and cross reactivity with other thyroid hormones/metabolites was analyzed by immune extraction from native and spiked serum in parallel using monoclonal antibodies (mAb) employed in the CLIA immune assays. Extracts bound to the mAb were subsequently analyzed by LC-MS-MS. No significant cross reactivity for the analytes indicated was detectable*.

* *Stable-isotopically-labeled THM used as internal standards in LC-MS/MS*.

## 3,5-T2 Mechanism of Action Involves Canonical TR Signaling and Rapid Direct Effects

### 3,5-T2 Action on Liver Is Accompanied by Potentially Adverse Effects on HPT-Axis and Heart

3,5-T2, in contrast to its structural isomers 3,3′-T2 and 3′,5′-T2, is an active ligand for the classical T3 receptors (TR), where it binds with relative high affinity (Table 4) and modulates transcriptional activity of TR, as indicated e.g., by rapid and powerful suppression of TSH *in vivo*, altered 3,5-T2 modulated gene expression *in vitro* and in several cellular models including anterior pituitary and liver (39, 81, 85–87). In addition to its action on TSH suppression in thyrotropes, 3,5-T2 also stimulates growth hormone production and secretion in somatotrophs (86). Apart from this work, elucidating thyromimetic effects similar to those exerted by T3 *in vivo* as well as in various experimental and cellular models, a group of researchers working with Goglia et al. over the last three decades presented evidence that 3,5-T2 might exert direct effects on mitochondria via interaction with proteins of the electron transport chain (e.g., cytochrome C oxidase) and with F0-ATPase [for review see (17)]. In addition to these actions, probably not mediated by mechanism related to the canonical TR nor its mitochondrial form, TRαp43 or TRαp22 (88), these authors claimed that 3,5-T2 might exert several beneficial actions on hepatic, muscular, and adipocyte tissues already at “low” concentrations. In their models apparently neither suppression of pituitary TSH production and secretion nor T3-typical adverse effects on the heart were observed at these doses used. Most of these studies were performed in severely hypothyroid, mostly rat models, and 3,5-T2 doses were administered acutely as well as chronically. Unfortunately, no information had been published on 3,5-T2 concentrations reached in the circulation or in those target tissues like liver, where beneficial effects such as antisteatotic activity and increased lipid metabolism were reported (15, 17, 28, 89, 90). The first evidence for rapid T3-independent effects of 3,5-T2 on mitochondrial activity and oxygen consumption was provided by Horst et al. (16), who observed rapid stimulation of oxygen consumption in isolated perfused livers, removed from hypothyroid rats. In their model, already low picomolar 3,5-T2 concentrations stimulated O_2_ consumption within 90 min, while T4 and T3 reacted more slowly and their effect could be blocked by adding 1 μM PTU to the perfusion medium, which acts as an inhibitor of DIO1, assumed to be the enzyme responsible for 3,5-T2 production. These authors studied several TH analogs with respect to stimulation of oxygen consumption in their model, in part complemented by analyses of stimulation of alpha-glycerophosphate-dehydrogenase, which is a typical biomarker for TH action on mitochondria. Moreno et al. (90) performed further studies on the time course and mechanisms involved in rapid stimulation of resting metabolic rate caused by THM. They observed that 3,5-T2 (25 μg/100 g body weight) exerted rapid effects in acutely treated hypothyroid rats while T3 reacted more slowly but with longer lasting effects compared to 3,5-T2. The protein synthesis inhibitor actinomycin D inhibited the effects of T3 similarly to observations made by Horst et al. (16) who showed that cycloheximide could block the T3 effect in their perfused liver model. Subsequently, the Naples teams, with investigator Goglia, added more endpoints to demonstrate rapid 3,5-T2 action, such as increased glucose consumption in muscle, which might involve sirtuin signaling (91). Several pathways involved in mediation of 3,5-T2 effects were subsequently studied using gene expression, functional, and proteomic readouts, especially for rat liver and muscle (92, 93) while comparable control data on potentially adverse effects of 3,5-T2 concentrations resulting in changes of liver and muscle metabolism were not systematically presented.

**Table 4:** Relative potencies of thyroid hormone metabolites at thyroid hormone receptors expressed as fold concentration required to displace T3 from binding to TRβ1 which is set as 1 (in bold) (corresponding to a K_d_ = 1 × 10^−10^ nM).

**Receptor**	**T4**	**T3**	**rT3**	**3,5-T2**	**Tetrac**	**Triac**	**References**
TRα1	14	2–20	–	–	–	3.9	(78)
TRβ1	3–22	**1**	300	–	33	3.6	(79, 80)
TRβ2	–	32	–	40–500	–	4.2	(81, 82)
pTRα_28_	–	3	–	–	–	–	(83)
Integrin α*νβ*3	3	–	–	–	–	–	(84)

*Published experimental data were obtained using various types of TH receptor preparations, Scatchard analyses or competition assays, resulting in IC-50 values. No systematic comparison was made using recombinantly expressed receptor under comparable binding conditions, ad different tissues, and species were used in competition experiments. Thus, only rough ranges are compiled here due to the limited comparability of experimental models*.

Controversial with respect to this set of observations, several other teams reported that administration of 3,5-T2 in rodent models (rat and mice, regular or high-fat diet, euthyroid or hypothyroid) dose-dependently resulted in significant suppression of the HPT axis as reflected by decreased TSH serum concentrations and/or pituitary beta-TSH transcript levels (6, 39, 87, 94, 95). Furthermore, thyromimetic effects typical for high circulating T3 were observed in the heart as analyzed by gene expression and/or morphological alterations suspicious of remodeling and fibrosis. Typically, those changes were then also accompanied by altered expression of T3 responsive genes in liver and other tissues. There have also been observations that 3,5-T2 interferes with the function of pancreatic islets altering glucagon and insulin expression and secretion (91, 96). Thus, it remains quite controversial whether 3,5-T2 would be an important lead compound in the development of antisteatotic drugs lacking unwanted adverse side effects at other TH responsive target tissues and reactions. The Naples teams also reported a pilot volunteer study, where two of the involved researchers dose escalated 3,5-T2 administration on themselves without observing unwanted side effects but reported on weight loss (97). This observation could not be supported by a randomized, placebo-controlled, double-blind, 4-week trial on male cardio-metabolic patients, who received a 50 mg daily dose of TRC-150094 (98). This is a 3,5-T2 mimetic agent previously shown to ameliorate metabolic risks in a high-fat diet obese rat animal model, where oxidative metabolism was significantly stimulated both in liver and skeletal muscle, and several metabolic pathways such as nitrogen, amino acid and sugar metabolism were shown to be altered using, among others, proteome analysis (99). Different from this promising animal experiment, the clinical trial provided no evidence for increased insulin sensitivity, changes in plasma free fatty acids, or intra-hepatic triglyceride content after administration of this agent (98). No further studies are available on this agent in rodents or humans.

### 3,5-T2 Accumulates in Tissues and Might Act by Intracrine Mechanisms

The adverse effects observed in rodent hearts have not been studied in more detail, and 3,5-T2 concentrations in cardiac rodent tissue were found to be below detection limits (100) in a study aiming to determine THM concentrations in rat heart. Pinna et al. (29) determined 3,5-T2 concentrations based on immunoassay under careful methodological precautions in rat brains, and found quite high 3,5-T2 concentrations, which differed among brain regions but were low in hypothalamus and pituitary. In the study by Jonas et al. (39), who administered two doses of 3,5-T2, only the higher one led to antisteatotic effects in the liver while both suppressed the HPT axis. That effect of 3,5-T2 on hepatic metabolism, especially lipid metabolism, might be due to a remarkable accumulation of 3,5-T2 in the liver, which so far had not been reported in appropriate experimental paradigms. Whether 3,5-T2 accumulation is an issue of increased import or decreased export remains to be studied, as the classical transporters for thyroid hormones such as MCT8, MCT10, OATP, and LATs typically show low transport activity for 3,5-T2 compared to the other T4, T3, or 3,3′-T2, as the structural isomer of 3,5-T2 (101, 102). Considering these controversial and—with respect to species analyzed and models employed—conflictings observations, the initial enthusiasm of developing 3,5-T2 related agents as anti-steatotic drugs, devoid of thyromimetic activity and side effects, remains questionable, and more research is definitely needed if this path should be further taken. The available data sets also strongly discourage use of 3,5-T2, highly popular in the body-building and wellness scene and freely available via the internet, as a sliming or energy-boosting drug.

## Data Availability Statement

All datasets generated for this study are included in the article/Supplementary Material.

## Ethics Statement

The Medical Ethics Committee of the relevant institutions approved each of the study protocols. The methods were performed in accordance with the approved guidelines. All included participants provided written informed consent in accordance with the Declaration of Helsinki. For the studies conducted at Georgetown University, the institutional review board approved the studies. The dialysis study was approved by the Institutional Review Boards of Hospital Divino Espírito Santo, Ponta Delgada, Açores-Portugal. The SHIP-TREND study was approved by the local ethics committee of the University of Greifswald and conformed to the principles of the declaration of Helsinki.

## Author Contributions

The manuscript has been drafted and written by JK. IL has performed the 3,5-T2 serum concentration measurements. KRe, ER, and KRi implemented the immune extraction studies with the 3,5-T2 monoclonal antibody. MP contributed the data and evaluation of the metabolome analysis. JA performed the dialysis study. MD and JJ planned, conducted, and evaluated the two clinical Jonklaas studies (53, 54). All authors have contributed parts of it, as well as read, edited, and seen the final submitted version.

### Conflict of Interest

The authors declare that the research was conducted in the absence of any commercial or financial relationships that could be construed as a potential conflict of interest.

## Footnotes

^1^Data are not shown for the 3,5-T2 serum profile after administration of T3 sulfate or T4/T3 combination therapy.

## Supplementary Material

Supplmentary Data Sheet 1 Serum concentrations of 3,5-T2 and 3-T1AM (relative to individual concentrations at t = 0 h) for the two kinetic studies administering Liothyronine (T3) to euthyroid volunteers (Figure 5C, upper panel) and hypothyroid patients (Figure 5C, lower panel).

Supplmentary Data Sheet 2 Serum concentrations of 3,5-T2 and 3-T1AM for each individual receiving Liothyronine in the two kinetic studies presented in Figure 6. Upper panel **(A)**: 12 euthyroid volunteers with blood drawn over 72 h; lower panel **(B)**: 18 hypothyroid patients with blood drawn over 8 h.
